# Understanding help-seeking intentions in male military cadets: An application of perceptual mapping

**DOI:** 10.1186/s12889-016-3092-z

**Published:** 2016-05-17

**Authors:** Sarah Bauerle Bass, Javier Muñiz, Thomas F. Gordon, Laurie Maurer, Freda Patterson

**Affiliations:** Department of Social and Behavioral Sciences, Temple University, College of Public Health, 1301 Cecil B. Moore Ave., Room 951, Philadelphia, PA 19122 USA; Department of Psychology, University of Massachusetts-Lowell, 113 Wilder St., Lowell, MA 01854-3059 USA; Department of Behavioral Health and Nutrition, University of Delaware, 026 North College Ave., Carpenter Sports Building, Newark, DE 19711 USA

**Keywords:** Perceptual Mapping, Males, Help-seeking, Communication, Perceived Control

## Abstract

**Background:**

Research suggests that men are less likely to seek help for depression, substance abuse, and stressful life events due to negative perceptions of asking for and receiving help. This may be exacerbated in male military cadets who exhibit higher levels of gender role conflict because of military culture.

**Methods:**

This exploratory study examined the perceptions of 78 male military cadets toward help-seeking behaviors. Cadets completed the 31-item Barriers to Help Seeking Scale (BHSS) and a component factor analysis was used to generate five composite variables and compare to validated factors. Perceptual mapping and vector modeling, which produce 3-dimensional models of a group’s perceptions, were then used to model how they conceptualize help-seeking.

**Results:**

Factor analysis showed slightly different groupings than the BHSS, perhaps attributed to different characteristics of respondents, who are situated in a military school compared to general university males. Perceptual maps show that cadets perceive trust of doctors closest to them and help-seeking farthest, supporting the concept that these males have rigid beliefs about having control and its relationship to health seeking. Differences were seen when comparing maps of White and non-White cadets. White cadets positioned themselves far away from all variables, while non-White cadets were closest to “emotional control”.

**Conclusion:**

To move these cadets toward help-seeking, vector modeling suggests that interventions should focus on their general trust of doctors, accepting lack of control, and decreasing feelings of weakness when asking for help. For non-White cadets a focus on self-reliance may also need to be emphasized. Use of these unique methods resulted in articulation of specific barriers that if addressed early, may have lasting effects on help-seeking behavior as these young men become adults. Future studies are needed to develop and test specific interventions to promote help-seeking among military cadets.

## Background

Men have a significantly lower life expectancy and higher disease morbidity compared to women in the United States [1]. One hypothesis to explain these poorer health outcomes is that they engage in riskier behaviors such as substance abuse, tobacco use, alcohol consumption and illegal drug use [2] and are less likely to seek help [3–5], which may be rooted in gender role socialization. Current theories posit that men and women learn gendered behaviors and attitudes through their respective cultures, impacting their attitudes, perceptions, and behaviors. The traditional values associated with masculinity, such as the need for control, emotional restraint, and independence, are often reinforced by media, family and friends, and cultural perceptions [6]. Men who have traditional beliefs about masculinity, and thus prescribe to strict gender role beliefs, are less likely to report negative symptoms, receive physical examinations, and are more likely to be diagnosed at later stages of disease [7–12]. The most recent analysis of the National Health Interview Survey notes that men are also more likely to say they do not have a usual place for health care, have not seen a doctor in the past year, and do not have health insurance [13].

In cultures where a masculinity ethos is predominant, such as the military, strength, courage, self-sufficiency, and resilience are promoted, all characteristics that act as barriers for military members when they need help [14]. The psychological effects of military service and combat can lead to depression, anxiety, and substance abuse, including the development of unhealthy drinking behaviors [15], which may be exacerbated if they believe that help-seeking goes against a masculine code for male military members. Many issues faced by male military members could be addressed through help-seeking behaviors, yet barriers to seeking help keep many soldiers away [16–18]. Although military cadets do not have the full range of experience of an enlisted soldier, they do become entrenched in the military culture, which may have lasting effects as they get older.

Actual and perceived stigma may serve to further promote these values among military populations [19, 20]. Anticipated or perceived stigma is the belief that one will be subjected to hostility, discrimination, or other negative attitudes if their help-seeking behaviors become known to others, while self-stigma occurs when an individual develops negative attitudes about himself for seeking help. Stigma theory posits that anticipated stigma may lead one to internalize these same attitudes and result in the adoption of these attitudes about oneself, thereby instigating self-stigma [21]. Zinzow et al. [22] found that stigma was one of the most frequently reported barriers to seeking mental health treatment among a general sample of active U.S. soldiers (80 % male) and a sample currently seeking mental health treatment (91 % male). Anticipated stigma was mentioned most commonly, yet self-stigma was also noteworthy among the general sample. Blais and Renshaw [19] found that although self-stigma and anticipated stigma were positively correlated with each other and negatively correlated with help-seeking behavior, the association between anticipated stigma and help-seeking was no longer significant after controlling for self-stigma, revealing that interventions focused on relieving self-stigma may be the most promising.

Zinzow et al. [22] found that concerns of confidentiality also keep many military members from seeking help, especially those in active service. Bulling et al. [23] suggested that military members who choose to seek help from chaplains rather than others likely do so because of the strict confidentiality that chaplains abide by. But the lack of exploratory studies focusing on military cadets and their help-seeking behaviors reflects a void in today’s literature. No current studies examine the combination of help-seeking and masculinity perceptions of military cadets, nor use novel analysis methods to understand how cadets conceptualize these variables as a way to provide insights into the barriers and facilitators of help-seeking behaviors. This study aimed to fill this gap by using factor analysis along with perceptual mapping and vector message modeling methods, which use multi-dimensional scaling processes to produce 3-dimensional models of complex cognitive and communication processes. These models assist with studying how framing effects, perceptions of risks/benefits, and attitudes toward risk contribute to cognitive and affective dimensions of decision-making. This method builds on the Galileo approach of Woelfel and Fink [24] and has a strong history as a mathematical modeling tool that can be used to identify optimum message or intervention strategies, based on the principles of increasing the attraction to, or repulsion from, particular concepts or attributes. Using this method, the main research question was to examine the major barriers and facilitators related to help-seeking in male military cadets and whether message strategies could be developed based on perceptual mapping methods.

## Methods

### Study design and participants

Study participants were a convenience sample of 78 male cadets aged 18-21 from a military college in the northeast region of the United States. Female cadets were excluded from the study because the goal of the study was to explore perceptions of male cadets toward help-seeking behaviors.

### Study procedures

The researchers were granted permission to attend an assembly at the military college with the purpose of inviting cadets to complete the study instrument. At the assembly, cadets listened to a review of the study and were told the criteria for inclusion. Inclusion criteria included being 18 years of age or older, being male, and being a cadet attending the college. Cadets who did not meet the inclusion criteria or did not wish to participate were asked to leave the room while those who were eligible were provided an informed consent form, which was read to them verbatim. Eligible and willing cadets signed the informed consent and completed the anonymous survey. All study materials and procedures were approved by the Temple University Institutional Review Board. Participants who completed the survey were given the opportunity to enter a lottery to win an iPod. Lottery entries were kept separate from informed consents to ensure anonymity.

### Instrumentation

Perceptions of barriers toward help-seeking behaviors were measured using 31-items from the *Barriers to Help Seeking Scale* (BHSS) [2, 25]. The BHSS presents five subscales: 1. The *Need for Control and Self-Reliance* (which reflects concerns with self-reliance and independence); 2. The *Minimizing Problem and Resignation* sub-scale (which addresses a number of barriers that prohibit people from seeking help. The minimization of the problem is determined by the degree to which the individual believes it is a legitimate problem); 3. The *Concrete Barriers and Distrust of Caregivers* sub-scale (which reflects barriers to help-seeking such as financial status, access to care, insurance, transportation, knowledge of available aid, and lack of trust of providers); 4. The *Privacy* sub-scale (which relates to emotional and physical vulnerability); and 5. The *Emotional Control* subscale (which relates to keeping one’s emotions to oneself and not expressing them to others). (For specific survey items, see Table 1.) The original scale is a Likert, five-point scale. In this study, participants responded to each survey item on an 11-point scale (0-10) where 0 represented strongly disagree and 10 represented strongly agree. This change in scale is based on the perceptual mapping process, described below. The demographic variables of age and ethnicity were also collected.

**Table 1:** Factors, survey items, factor loading and percentage of variance

Factor and Items	Factor Loading	% Variance
*Factor 1: Self*-*reliance*		35.9 %
It would seem weak to ask for help	.773	
Asking for help is surrendering authority over my life	.751	
I feel better about self knowing I didn’t need help	.666	
I like to be in charge of everything in my life	.607	
I think less of myself for needing help	.602	
Nobody knows about my problems	.590	
I do not want to appear weaker than my peers	.588	
I like to make my own decisions and not be influenced by others.	.575	
Don’t like people telling me what to do	.533	
*Factor 2*: *Resignation*		9.4 %
I’d prefer to suck it up rather than dwell on my problems	.821	
Problems like this are a part of life; they’re just something you have to deal with	.796	
I’d prefer to wait until I’m sure the health problem is a serious one	.793	
The problem wouldn’t be a big deal; it would go away in time	.753	
I wouldn’t want to overreact to a problem that wasn’t serious	.718	
The problem wouldn’t seem worth getting help for	.686	
*Factor 3*: *Privacy*		7.3 %
I don’t want some stranger touching me in ways I’m not comfortable with	.784	
I don’t like taking my clothes off in front of people	.783	
I wouldn’t want someone of the same sex touching my body	.667	
Sharing personal medical information is embarrassing	.621	
I don’t like feeling controlled by others	.502	
*Factor 4*: *Emotional Control*		5.9 %
I’d rather not show people what I’m feeling.	.847	
I don’t like to get emotional about things.	.824	
I don’t like to talk about feelings.	.746	
I wouldn’t want to look stupid for not knowing how to figure this problem out.	.666	
Privacy is important to me, and I don’t want other people to know about my problems	.489	
*Factor 5*: *Structural Barriers to Help*-*Seeking*		5.6 %
I wouldn’t know what sort of help was available	.854	
I would have real difficulty finding transportation to a place I can get help	.849	
Financial difficulties would be an obstacle to getting help	.740	
A lack of health insurance would prevent me from asking for help	.653	
People typically expect something in return when they provide help	.484	
I don’t trust doctors and other health professionals	.408	
TOTAL VARIANCE EXPLAINED		64.1 %

### Data analysis

To generate BHSS constructs specific to this population of military cadets, and to reduce the data for processing in the perceptual maps, a component factor analysis of the 31-items from the BHSS was conducted to assess fit of the five factor model to the study data. Varimax rotation with Kaiser normalization was used and survey items with eigenvalues >1 were extracted; items loading with > 0.4 were retained [26]. These analyses were done using SPSS version 23.0.

**Fig. 1:**
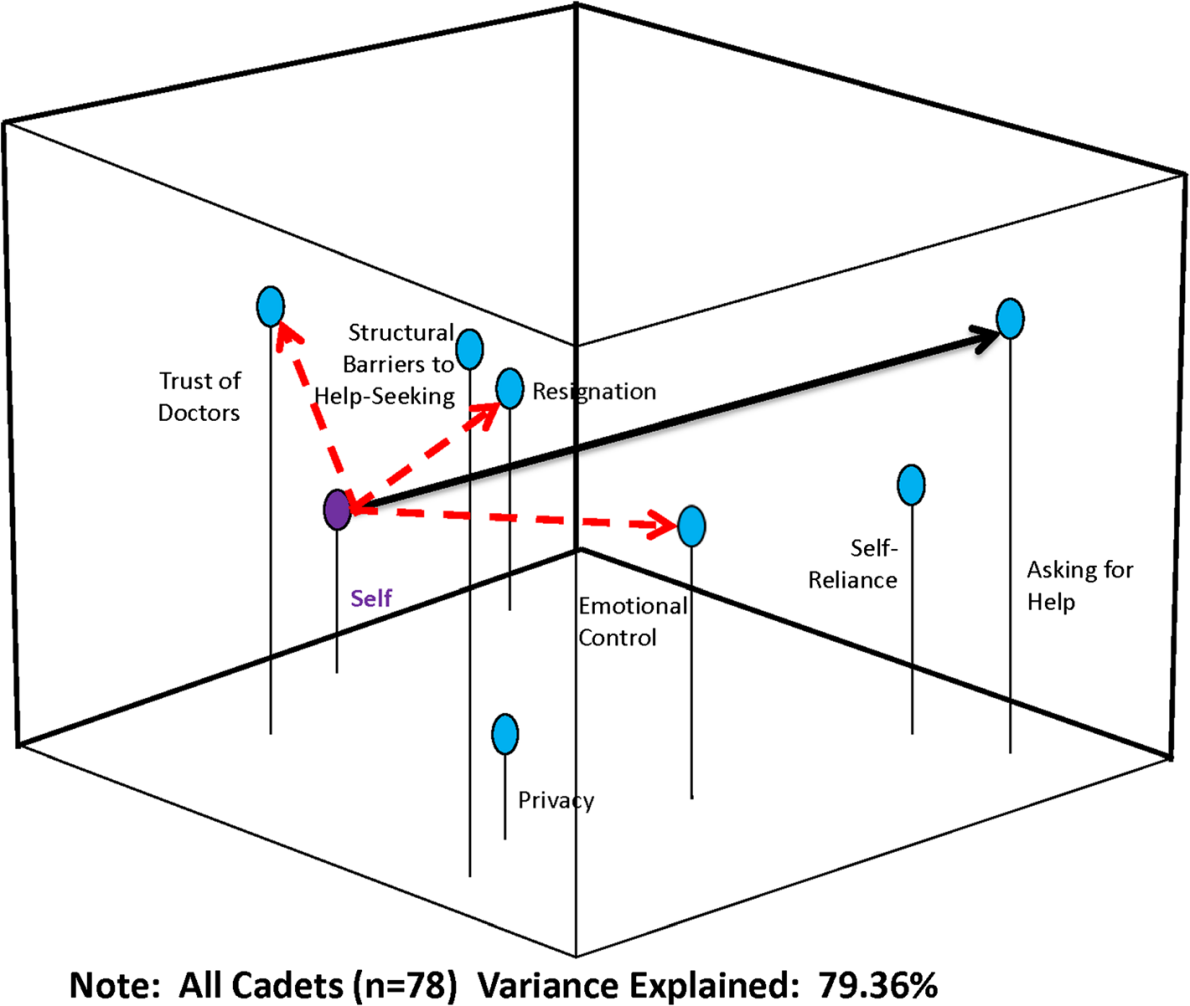
Perceptual Map with Message Vectors to “Asking for Help”: All Cadets

**Fig. 2:**
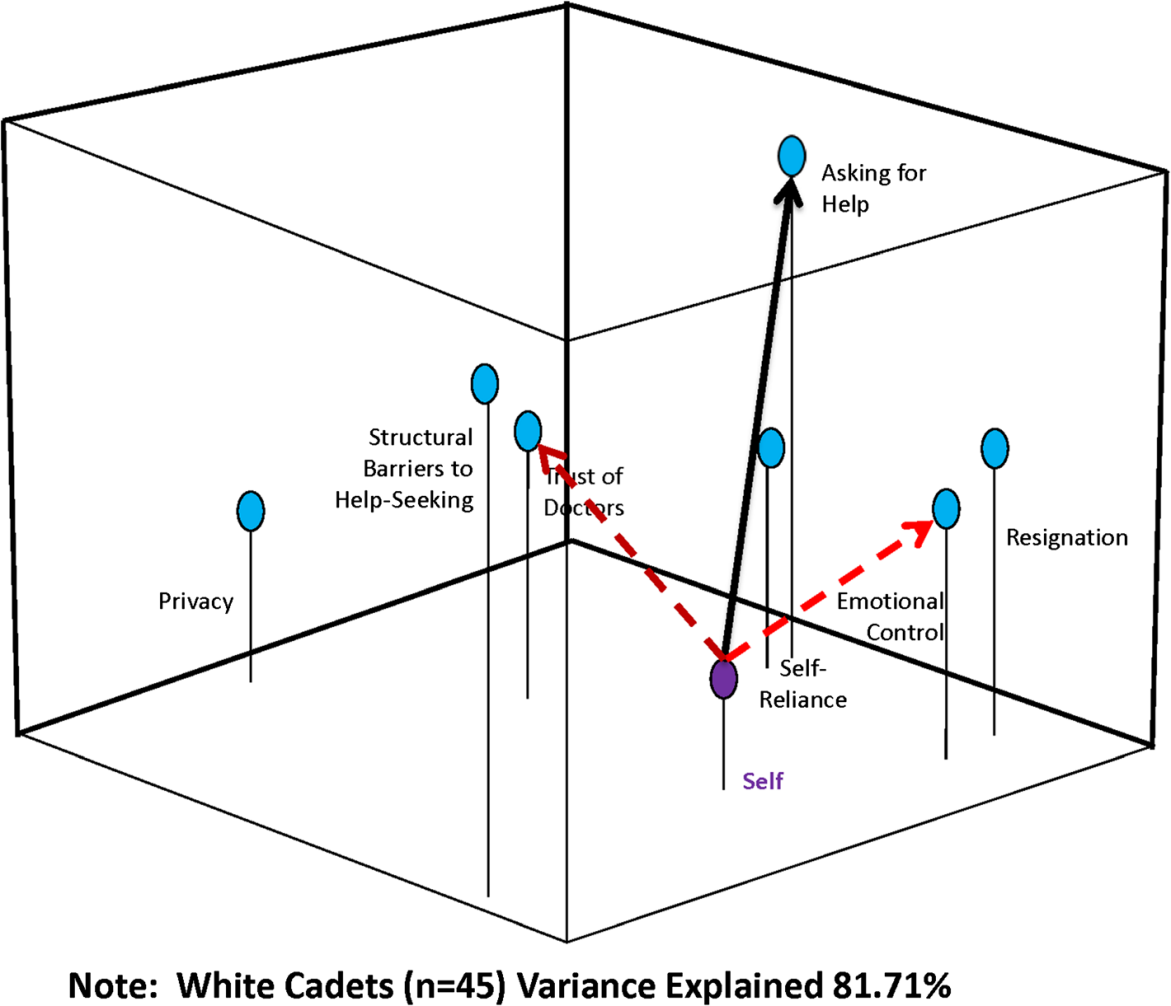
Perceptual Map with Message Vectors to “Asking for Help”: White Cadets

**Fig. 3:**
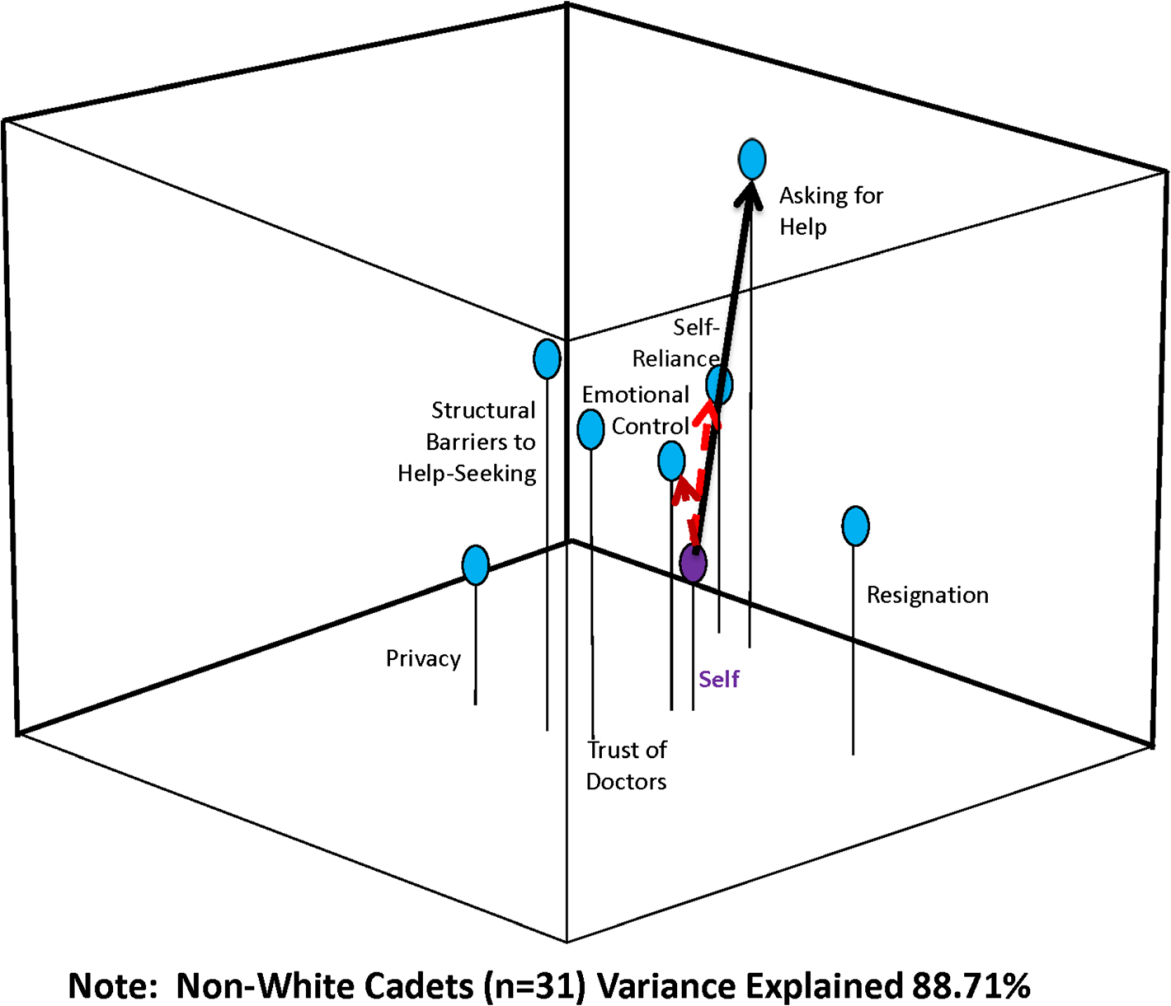
Perceptual Map with Message Vectors to “Asking for Help”: Non-White Cadets

Perceptual mapping and vector message modeling techniques were used to map the spatial relationship of constructs related to help-seeking behavior and develop strategies for possible targeted messages. These methods are used extensively in marketing and advertising, and have been used to evaluate a number of public health decisions by the authors [27–30]. They use multidimensional scaling (MDS), which produce a three-dimensional graphic display of how participants perceive relationships among a set of elements (e.g. risks and benefits). The resulting maps (see Figs. 1, 2 and 3) show how cadets perceive help-seeking constructs relative to each other and relative to “self”. In a perceptual map, “self” can be positioned in the model either as an individual (if the map is based on only one person) or as a group/sample average when data are combined for multiple respondents. The ability to construct and analyze maps for segmented representative subgroups is critical for extracting information needed for targeting and tailoring messages [27–30]. (Methodological details about perceptual mapping techniques used in this study are available at: https://sites.temple.edu/turiskcommlab/)

#### Development of perceptual maps

The mapping method uses surveys that require subjects to rate the extent to which they associate specific elements with each other (based on similarities and differences of perceived association). Unlike other mental mapping procedures that require the respondent to make complex overall judgments, perceptual mapping only requires subjects to judge the individual items; the software then puts these component parts together as a whole model, making the instrument easy for participants to use. The result is a graphic display of the data structure rather than the typical statistical summary tables associated with survey research.

To construct the perceptual maps, we have developed software based on the metric MDS program Galileo [24]. This program converts the scaled judgments into distances used in the mapping. Input associations among the factors are derived from the inter-item correlations of all elements, where the absolute values of the Pearson product-moment correlations are converted to a 0-10 scale base. Thus, all distance matrix input data are on the same 0-10 scale. Input values are also “reflected” so that more important elements appear closer to the “aggregate self,” while those judged less important are farther away [27].

In the last step, the software performs a metric multidimensional scaling analysis and produces graphic arrays of the distances among the elements. The percentage of variance accounted for by the analysis is provided as an assessment of the explanatory value of each map (see Figs. 1, 2 and 3). The resulting map displays the risk/benefit elements relative to each other, and to the “aggregate self”. Ultimately, the maps provide a snapshot of the respondents’ conceptualization of the situation and reveal the relative importance of different elements [27] for the cadets as a whole, and segmented by race/ethnicity. This method has been found to be highly valid; if you enter distances between American cities into the program, a map that shows the cities in proper relative positions to each other is constructed with very little error (SSTRESS = .003) [31]. We, and others, have shown it to also be valid using varying numbers of participants [27–30, 32–36], and in fact a perceptual map can be developed for an individual.

After the perceptual map is created, vector message modeling methods are used to understand how to “move” individuals or concepts/attributes within the perceptual space toward a desired position, decision, behavior, or attitude; in this case how to effectively communicate with cadets about the benefits of help-seeking. Because perceptual maps are mathematical models, vector analyses can be used to determine optimum associations to emphasize in a message or intervention in order to change the positioning of elements in the perceptual space (see lines/arrows in Figs. 1, 2 and 3). Once the perceptual map is produced reflecting how the group conceptualizes the relationships among the concepts/attributes, it shows which attributes (positive and negative) the group most closely associates with the concept-as a simultaneously interacting mix that includes the “Self.” The vector message design analysis can then be done by specifying which element (or elements) in the space should be moved, and where in the space it is to be repositioned. Mathematically, this defines the “target vector”.

Vector modeling methods can then help the researcher understand how to “move” individuals within the perceptual space toward the desired decision or attitude. This can help develop message “parsimony”, by only focusing on those variables most important to the desired change, rather than employing the “kitchen sink” method of message/intervention development. If too many concepts are emphasized, the true motivators for change may get lost in a message or intervention that is too complicated. While the process of crafting the messages and interventions remains subjective, vector modeling methods provide the researcher and program developers an empirical basis on which to select message elements to include that are most likely to improve outcomes. In this study, the target vector was “help-seeking”, indicating the need to “move” cadets towards being more accepting of help-seeking behaviors. Results can then be used to create highly targeted interventions.

## Results

### Factor analysis

A component factor analysis was used to validate the five factors of the BHSS, as described above. Factor loading was very similar, with only two of the 31 items not loading on the same factor. The item “I don’t like feeling controlled by other people” loaded on Privacy factor (instead of the Control and Self-reliance factor) and the item “Privacy is important to me and I don’t want other people to know about my problem” loaded on the Emotional Control factor (instead of the Privacy factor). The published variance explained by the BHSS is 57.2 % [2]; ours yielded an explained variance of 64.1 %. A full listing of these constructs and the loading of each of the survey items can be found in Table 1.

### Descriptive results

Over half of the 78-person sample was White (*n* = 45; 58 %). Of the non-Whitess (*n* = 31; 40 %), 12 % were African American (*n* = 9), 10 % were Asian/Pacific Islander (*n* = 8), 8 % were Latino/Hispanic (*n* = 6) and 10 % self-reported being an “other” ethnicity (*n* = 8). Two respondents (3 %) did not provide racial data. All respondents were between the ages 18 and 21: 23 % (*n* = 18) were aged 18, 35 % (*n* = 27) were 19, 26 % (*n* = 20) were 20, and 17 % (*n* = 13) were aged 21.

On a scale of 0-10, cadets self-reported relatively high levels of resignation (*M* = 6.39, SD = 2.44), emotional control (*M* = 6.66, SD = 2.58), and self-reliance (*M* = 5.91, SD = 2.14). Lower scores were evident for privacy (*M* = 5.07, SD = 2.27) and structural barriers to help-seeking (*M* = 4.43, SD = 2.60). To specify trust of doctors and help-seeking, the items “I don’t trust doctors and other health professionals” (*M* = 2.72, SD = 3.12) and “Asking for help is like surrendering authority over my life” (*M* = 3.74, SD = 3.47) were used separately in the perceptual mapping analysis.

### Perceptual mapping results

The perceptual mapping software produced three-dimensional maps displaying the composite variables in relation to *self*, the group mean. These were constructed for the whole sample (*n* = 78) and for White (*n* = 45) and non-White (*n* = 31) cadets separately. As shown in Fig. 1, cadets see “trust of doctors” as “closest” to them and “asking for help” farthest away. Conceptually they are also grouping “self-reliance” with “asking for help.” The cumulative percentage of variance explained by the map is 79.36 %, indicating a high degree of consistency for the factor relationships.

Figure 2 displays the relationship of factors for White cadets. “Trust of doctors” is farther away for this group, while “self-reliance” is closer. “Asking for help” is still, however, farther away conceptually. The cumulative percentage of variance explained by this map is 81.71 %. Figure 3 displays the relationships for non-White cadets. In this group, “emotional control” is closest to self and “asking for help” is not as far away. The cumulative percentage of variance explained in this case is 88.71 %.

The vector analysis is denoted by the solid and dotted arrows in Figs. 1, 2 and 3. The solid line illustrates where in the three-dimensional space you want the group to move (toward “asking for help”) and the dotted arrows show which concepts would need to be emphasized in a message strategy or intervention to move the group toward “asking for help”.

## Discussion

The aim of this study was to assess the perceptions of help-seeking behavior in a sample of male military cadets. The main findings were that cadets reported a limited willingness to seek help and interventions would need to focus on their feelings of emotional control, as well as their self-concept of being resigned to minimizing problems. In the overall group, since the group believes that asking for help indicates weakness (indicated by its distance to self), an intervention strategy to increase cadets’ willingness to seek help would be important. Based on the vector analysis, to effectively reposition cadets in the space, interventions should emphasize their trust of doctors, address their feelings of resignation and focus on help-seeking as a way to maintain emotional control. Messages would need to address the feeling of “losing control” in a situation, perhaps emphasizing the control they maintain in a help-seeking situation, and how being strong and self-reliant is in concert with taking care of oneself.

Racial differences in perceptual maps between White and non-White cadets were also found, indicating slightly different message strategies for an intervention. Non-Whites conceptualized having emotional control closer to themselves than White cadets and addressing their concept of self-reliance would be important in an intervention. Whites were instead less likely to conceptualize any of the variables as close to them and an intervention would need to focus more on their overall trust of doctors and their feelings of emotional control. For non-White cadets, “trust of doctors” does not seem to be an important concept to them and its emphasis may not be salient to them in an intervention. Instead, the messages focusing on “self-reliance” and “emotional control” will be more important to move this group toward help-seeking. While caution should be applied to these findings based on the diverse nature of the non-White group, it suggests that interventions aimed at increasing help-seeking may need slightly different message framing to encourage White and non-White cadets. Overall, it is clear from this analysis that military cadets accept the masculine ethos and strict gender role stereotype, and successful interventions would have to address these perceptions to encourage and ultimately increase willingness to seek help.

This has direct implications on behavior of adult military members. Recent studies of veterans returning from Iraq and Afghanistan indicate that this reluctance to seek help persists. Quartana, Wilk, Thomas et al. [37] found that more than half of those returning with mental health issues did not seek help, illustrating the significant barriers to help-seeking and potential negative consequences. Other studies have found similar reluctance in help-seeking [20, 38]. Though these studies focused on mental health services and mental health stigma specifically, in a systematic review Sharp and colleagues [39] note that other factors besides stigma should be considered in interventions as stigma alone does not explain lack of help-seeking in military personnel. Results from the perceptual mapping and vector modeling analysis converge with these findings. Cadets see having control of their emotions as important to their self-identity. Being perceived as emotional or unable to solve problems without outside help is viewed as a sign of weakness, supporting the concept of self-stigma as an important barrier to help-seeking [19]. Self-reliance is also an important concept to emphasize in a message strategy*,* reflecting the fact that cadets believe it an important concept and that they should have control over their own decisions and lives. Overall, in terms of understanding how cadets may think about health protecting behaviors, it is clear that bringing them closer to the concept of help-seeking would be key in assisting them with being open to seeking and acting on health advice. Our perceptual mapping analysis supports this. Using messages that promote masculine emotions and the acceptance of one’s limitations or weaknesses by promoting their self-reliance and control of situations will, in theory, help to move cadets closer to the help-seeking target. Appropriately emphasizing these concepts, in conjunction with concepts that address perceptions of weakness, control and self-reliance should also “pull” the group closer to help-seeking. This might be an important intervention strategy to help cadets feel comfortable seeking and asking for health related information or services and not equating that seeking with feeling weak or modest. One caveat that we identified, however, is that non-White cadets may need slightly different messaging. Studies indicate that non-Whites have a higher mistrust of the medical system, stemming the historical legacy of medical mistreatment such as the Tuskegee syphilis experiment [40–42]. These feelings may hold true to younger men of color as well, making the emphasis on trust of doctors not as salient.

Overall, rethinking the way men are being reached with health information is important to the development of better health interventions, as are improved ways of encouraging men to take advantage of preventative and screening services that are directed towards men. Having better tools to “look into the mind” of enlisted men is critical if we are to more effectively identify and anticipate their needs and concerns. The literature has shown that military personnel are at increased risk for suicidal ideation, alcohol and substance abuse, unprotected sex, and other risky behaviors [43, 44]. Concomitantly, the influx of veterans returning to the United States has demanded increased health care services for the mentally and physically wounded. It is clear that we must increase our efforts to help all military personnel optimize their help-seeking behaviors in order to take advantage of these services.

### Strengths and limitations

The findings of this study provide insight into how health messages can be targeted to promote intention to seek help among military college cadets. This study also has certain limitations. The sample was a convenience sample of 78 cadets, which may not represent the whole military cadet population. Permission to enter the college and have access to the cadets was provided by the superintendent of the college, which may have influenced which cadets did or did not participate. There also were more White than non-White cadets and the non-White group was highly diverse, so the analysis comparing these groups may not be a valid representation of real differences in help-seeking perceptions. In addition, because the sample was drawn from college-aged cadets, the factor structure may vary when used in an older population. As age increases, structural barriers may be more of an influential factor toward help-seeking, such as health insurance and transportation, which may affect older groups differently compared with cadets who would require some level of insurance coverage to attend school. In addition, the factor analysis phase of this study used an eigenvalue of 1 as a cutoff for construct development, and while this cutoff is often used [45, 46], there is debate about this approach being too conservative or limiting. In addition, while our confirmatory factor analysis was a good fit with the BHSS validated factor analysis, two items did load differently. It could be that because of sample characteristics and the inherent differences between male military cadets and more typical college-aged males, the scale did not as adequately assess barriers to help-seeking in this group. However, our analysis yielded a larger percent variance explained, indicating that our item loadings enhanced the power of the results.

## Conclusions

Research literature supports the need to improve outreach to men to use health services to address disparities in health outcomes, including morbidity and mortality of many health issues [47]. Recent analysis indicated that men with strong masculinity beliefs are half as likely as men with more moderate masculinity beliefs to receive preventive healthcare [48]. In order to more effectively reach males, more targeted efforts are needed to expand their access to and use of available health information, and address their unique needs to ensure help-seeking is valued and encouraged. This study illustrated the unique challenges military culture creates in addressing barriers to help-seeking in young men.

Developing salient interventions may be especially challenging in a male cadet population, such as the one studied. Future studies are needed to develop and test specific health messages to promote trust among cadets so they feel comfortable seeking help. Further studies are also needed to understand perceptions of active-duty members, to see if barriers to help-seeking persist. Comparing the perceptions of cadets, who plan to continue their military career, with active-duty members, will identify important distinctions between the two groups and how interventions may be developed to bridge the two. Using novel methodologies like perceptual mapping and vector modeling helps identify barriers and facilitators of help-seeking, and can identify specific message strategies for interventions that effectively modify cadet perceptions in ways that will promote their help-seeking behaviors.

## Ethics approval and consent to participate

This study was approved by the Temple University Institutional Review Board, number 20337.

## Consent for publications

Not applicable.

## Availability of data and materials

If readers are interested in the data on which this manuscript is based, please contact the lead author for access: Sarah Bauerle Bass, sbass@temple.edu

## Abbreviations

BHSS: behavior health seeking scale
MDS: multi-dimensional scaling
